# Noninvasive Staging of Lymph Node Status in Breast Cancer Using Machine Learning: External Validation and Further Model Development

**DOI:** 10.2196/46474

**Published:** 2023-11-20

**Authors:** Malin Hjärtström, Looket Dihge, Pär-Ola Bendahl, Ida Skarping, Julia Ellbrant, Mattias Ohlsson, Lisa Rydén

**Affiliations:** 1 Division of Oncology Department of Clinical Sciences Lund University Lund Sweden; 2 Division of Surgery Department of Clinical Sciences Lund University Lund Sweden; 3 Department of Plastic and Reconstructive Surgery Skåne University Hospital Malmö Sweden; 4 Department of Clinical Physiology and Nuclear Medicine Skåne University Hospital Malmö Sweden; 5 Department of Surgery Skåne University Hospital Malmö Sweden; 6 Department of Astronomy and Theoretical Physics Lund University Lund Sweden; 7 Centre for Applied Intelligent Systems Research Halmstad University Halmstad Sweden; 8 Department of Surgery and Gastroenterology Skåne University Hospital Malmö Sweden

**Keywords:** breast neoplasm, sentinel lymph node biopsy, SLNB, noninvasive lymph node staging, NILS, prediction model, multilayer perceptron, MLP, register data, breast cancer, cancer, validation study, machine learning, model development, therapeutic, feasibility, diagnostic, lymph node, mammography images

## Abstract

**Background:**

Most patients diagnosed with breast cancer present with a node-negative disease. Sentinel lymph node biopsy (SLNB) is routinely used for axillary staging, leaving patients with healthy axillary lymph nodes without therapeutic effects but at risk of morbidities from the intervention. Numerous studies have developed nodal status prediction models for noninvasive axillary staging using postoperative data or imaging features that are not part of the diagnostic workup. Lymphovascular invasion (LVI) is a top-ranked predictor of nodal metastasis; however, its preoperative assessment is challenging.

**Objective:**

This paper aimed to externally validate a multilayer perceptron (MLP) model for noninvasive lymph node staging (NILS) in a large population-based cohort (n=18,633) and develop a new MLP in the same cohort. Data were extracted from the Swedish National Quality Register for Breast Cancer (NKBC, 2014-2017), comprising only routinely and preoperatively available documented clinicopathological variables. A secondary aim was to develop and validate an LVI MLP for imputation of missing LVI status to increase the preoperative feasibility of the original NILS model.

**Methods:**

Three nonoverlapping cohorts were used for model development and validation. A total of 4 MLPs for nodal status and 1 LVI MLP were developed using 11 to 12 routinely available predictors. Three nodal status models were used to account for the different availabilities of LVI status in the cohorts and external validation in NKBC. The fourth nodal status model was developed for 80% (14,906/18,663) of NKBC cases and validated in the remaining 20% (3727/18,663). Three alternatives for imputation of LVI status were compared. The discriminatory capacity was evaluated using the validation area under the receiver operating characteristics curve (AUC) in 3 of the nodal status models. The clinical feasibility of the models was evaluated using calibration and decision curve analyses.

**Results:**

External validation of the original NILS model was performed in NKBC (AUC 0.699, 95% CI 0.690-0.708) with good calibration and the potential of sparing 16% of patients with node-negative disease from SLNB. The LVI model was externally validated (AUC 0.747, 95% CI 0.694-0.799) with good calibration but did not improve the discriminatory performance of the nodal status models. A new nodal status model was developed in NKBC without information on LVI (AUC 0.709, 95% CI: 0.688-0.729), with excellent calibration in the holdout internal validation cohort, resulting in the potential omission of 24% of patients from unnecessary SLNBs.

**Conclusions:**

The NILS model was externally validated in NKBC, where the imputation of LVI status did not improve the model’s discriminatory performance. A new nodal status model demonstrated the feasibility of using register data comprising only the variables available in the preoperative setting for NILS using machine learning. Future steps include ongoing preoperative validation of the NILS model and extending the model with, for example, mammography images.

## Introduction

Breast cancer is the most frequently diagnosed cancer worldwide. Despite its generally favorable prognosis [1], the focus on the quality of life for affected patients is becoming increasingly important. For the last 2 decades, sentinel lymph node biopsy (SLNB) has been the standard surgical procedure for evaluating axillary status in patients with breast cancer and clinically node-negative (cN0) status [2]. The SLNB procedure causes less postoperative morbidity than axillary lymph node dissection; however, it is still associated with lymphedema, arm pain and numbness, and reduced quality of life [3]. Furthermore, in 70% to 80% of cases [4], SLNB will prove negative, without cancer cells in the sentinel lymph nodes, and surgical axillary intervention will have no therapeutic benefit.

Multiple recent studies have presented prediction models for noninvasive staging of axillary nodal (N) status with the long-term aim of replacing SLNB for subgroups of patients with breast cancer [5-17]. Only routinely and preoperatively available data should be used for a feasible noninvasive diagnosis of axillary N status aimed at clinical implementation. A limitation of the published models is that they include postoperative variables from surgical specimens, including pathological tumor size [10,14], estrogen receptor (ER) status [5,7,13,16], progesterone receptor (PR) status [5,7], human epidermal growth factor receptor 2 (HER2) status [5,7,10,16], proliferation index Ki67 value [5,7,13], Nottingham histological grade (NHG) [5,7,8,12], histological type [5,7,8,12], and lymphovascular invasion (LVI) [6,7,11].

ER, PR, HER2, and Ki67 showed moderate to very good concordance between core needle biopsy (CNB) and surgical specimens [18]. Therefore, these variables have potential as preoperative predictors of lymph node status. Similarly, NHG and histological type showed concordance rates of >70% [19] and >80% [20], respectively, for the same comparison. However, LVI is challenging to evaluate on preoperative CNB because of the limited amount of tissue sample, and a high failure rate of 30% has been reported [21]. Along with tumor size, LVI status is the most important clinicopathological predictor of N status [22]. Although preoperative evaluation of LVI remains a challenge, an accurate preoperative assessment of LVI is needed to predict the N status.

Imaging of the breast and axilla can be used to assess preoperative tumor size and extract other features related to the N status. Standard imaging modalities in the diagnostic workup of breast cancer are mammography and ultrasound (US) of the breast and axilla; therefore, data from these imaging modalities can be obtained routinely. Several models have been developed using US features [5,10,11,16,17]. However, US is operator dependent; therefore, it is not reproducible, limiting its utility in prediction models. In addition, prediction models using other imaging modalities or combinations, such as US and magnetic resonance imaging (MRI) [9], positron emission tomography combined with US [13], MRI [14], contrast-enhanced spectral mammography (CESM) [15], and US combined with computed tomography [16], lack clinical feasibility.

Nomograms have been developed based on postoperative, nonimaging, and pathological data. Li et al [8] showed an internal validation area under the receiver operating characteristic curve (AUC) of 0.718 (95% CI 0.714-0.723) when predicting lymph node metastasis including tumor size, NHG, and histological type. The discriminatory performance of the Memorial Sloan-Kettering Cancer Center nomogram [22] for the prediction of sentinel lymph node metastasis, developed based on 3786 patients, decreased significantly from an AUC of 0.75 in the internal validation to an AUC of 0.67 (95% CI 0.63-0.72) when externally validated in a Dutch population (n=770) [23]. Furthermore, the Skåne University Hospital nomogram [6], a logistic regression model based on 800 patients in Lund, Sweden, aiming to predict negative sentinel lymph nodes, had an internal validation AUC of 0.74 (95% CI 0.70-0.79). The nomogram was temporally (n=1318) and geographically (n=1621) externally validated with an AUC of 0.75 (95% CI 0.70–0.81) and an AUC of 0.73 (95% CI 0.70–0.76), respectively [24].

In 2019, Dihge et al [7] predicted axillary N status in patients with cN0 breast cancer using a multilayer perceptron (MLP) model for noninvasive lymph node staging (NILS) based on 15 clinical and postoperative pathological predictors. The NILS concept includes logistic regression and machine learning models for noninvasive staging of the axilla, aiming at a web interface implementation to be used in clinical practice. Similar to previous N prediction models, pathological tumor size and LVI were the top-ranked predictors in the original (MLP) NILS model [7]. Training and internal cross-validation were performed on the same 800 patients as in the study by Dihge et al [6] and provided a prediction of the disease-free axilla. In addition, the possible clinical benefit of using the model to identify patients who were least likely to benefit from SLNB was assessed. Surgical axillary lymph node staging could have been avoided in 27% of patients, given a false-negative rate (FNR) of 10%, corresponding to the accepted FNR for SLNB [25]. Although the benefit of replacing logistic regression with machine learning in clinical prediction models is not specified [26], the MLP model outperformed the multivariable logistic regression model, given its discriminatory performance.

This study primarily aimed to externally validate the original NILS model presented in 2019 [7] and develop a new N model in a large population-based cohort of routinely collected data from the Swedish National Quality Registry for Breast Cancer (NKBC). In addition, it secondarily aimed to develop an LVI model and assess how the overall predictive performance of the N model was affected by applying the LVI model for missing values. To the best of our knowledge, this is the first LVI model to be incorporated into an N model. This study was conducted in accordance with the Transparent Reporting of a multivariate prediction model of Individual Prognosis Or Diagnosis (TRIPOD) to develop and validate prediction models [27].

## Methods

### Study Population

Three data sets with nonoverlapping populations were used for model development and evaluation. The inclusion criteria for all 3 cohorts were female patients with invasive primary breast cancer and cN0 axilla scheduled for primary surgical treatment, with excision of the breast tumor by total mastectomy or partial mastectomy and axillary staging by SLNB. In addition, the exclusion criteria for the 3 cohorts were male sex, previous ipsilateral breast or axillary surgery, bilateral cancer, previous neoadjuvant therapy, ductal carcinoma in situ only, missing pathological-anatomical diagnosis tumor size, tumor size >50 mm, a tumor growing into the chest wall or skin, metastatic disease (stage 4 breast cancer), patients with clinically node-positive disease, and missing or incongruent data for axillary surgery or lymph node status.

**Figure 1:**
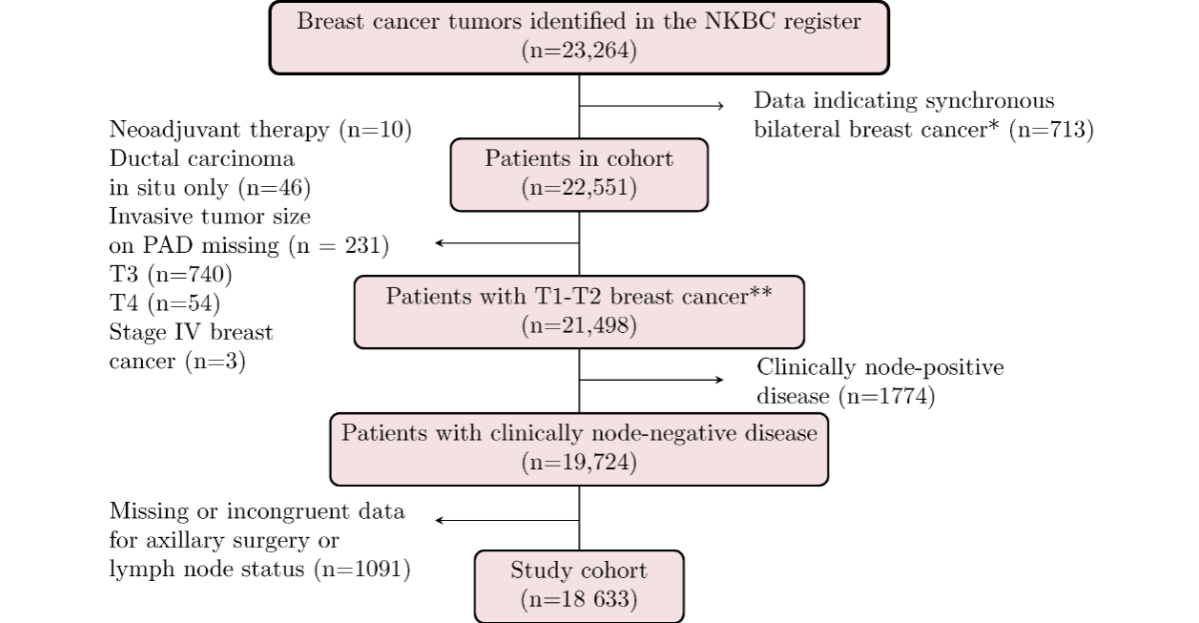
Patient selection for cohort II. *Including records with the same information on age, mode of detection, hospital, and date of diagnosis but with different laterality. **Note that 31 patients were excluded by 2 of the 6 criteria in this step. NKBC: Swedish National Quality Register for Breast Cancer; PAD: pathological-anatomical diagnosis.

The 3 data sets originated from different periods. Data set 1 (n=995) comprised consecutive patients diagnosed with primary breast cancer at Skåne University Hospital Lund, Sweden, between January 2009 and December 2012. Data were extracted from the medical records and pathology reports, with a final cohort size of 761 (Multimedia Appendix 1). For data set 1, a quality assessment scheme was used to ensure accurate histopathological reporting and internal quality control of the retrieved data from the medical records. Data set 2 (n=23,264) was a large population-based cohort of a breast cancer registry for external validation and development of a new N model. It consisted of patients with primary breast cancer from all breast cancer treatment units in Sweden included in the NKBC registry from 2014 to 2017, with a final cohort size of n=18,633 (Figure 1). Löfgren et al [28] examined the data quality of NKBC in 2019 and reported high validity and coverage of 99.9% between 2010 and 2014. Data set 3 (n=598) comprised consecutive patients with primary breast cancer surgically treated in Malmö or Helsingborg, Sweden, between 2019 and 2020, respectively. Data were, similar to those of data set 1, extracted from medical records and pathology reports. The final cohort size was 525 patients (Multimedia Appendix 2). The data extraction for cohort III was validated and monitored by an independent researcher according to a specific quality assurance protocol [29]. The sample size calculation for validating the NILS concept has been published previously [29].

### Outcomes

The following 2 outcomes were assessed: pathological N status (node-negative [N0] vs node-positive [N+] disease) and pathological LVI status (LVI-positive vs LVI-negative disease). Lymph node involvement was defined as metastatic infiltration of >0.2 mm in the lymph nodes; therefore, patients with only N micrometastasis were included in the study and categorized as N+. LVI positivity was defined as the presence of tumor cells within endothelium-lined lymphatic channels or blood vascular vessels [30]. A board-certified specialist in clinical pathology assessed both outcomes on surgical breast specimens according to the national guidelines for pathology [30].

### Data Availability and Preprocessing

The original NILS model [7] included the following variables available preoperatively: age at diagnosis, BMI, tumor laterality, mode of detection (mammographic screening or symptomatic presentation), menopausal status, tumor localization (centrally or 1 to 12 o’clock position), and variables assessed on surgical breast specimens (ie, largest pathological tumor size, tumor multifocality assessed by pathology, histological type, NHG, LVI status, ER status, PR status, HER2 status, and Ki67 labeling index). The inclusion of tumor characteristics and lymph node status in the contralateral breast and axilla violated the assumption of independent samples, and patients with bilateral tumors were excluded (Figure 1). Although the information on LVI status was missing in cohort II, a separate prediction model for LVI status was developed in cohort I because of its importance in predicting N status [7,22]. All variables were defined and preprocessed as described by Dihge et al [7], except for the histological type. In cohorts I and II, the histological type was categorized into the following 3 groups: no special type, lobular, and other or mixed. In cohort III, data on other or mixed histological type were regrouped, and the mixed histological type was set as missing.

### Study Design

**Figure 2:**
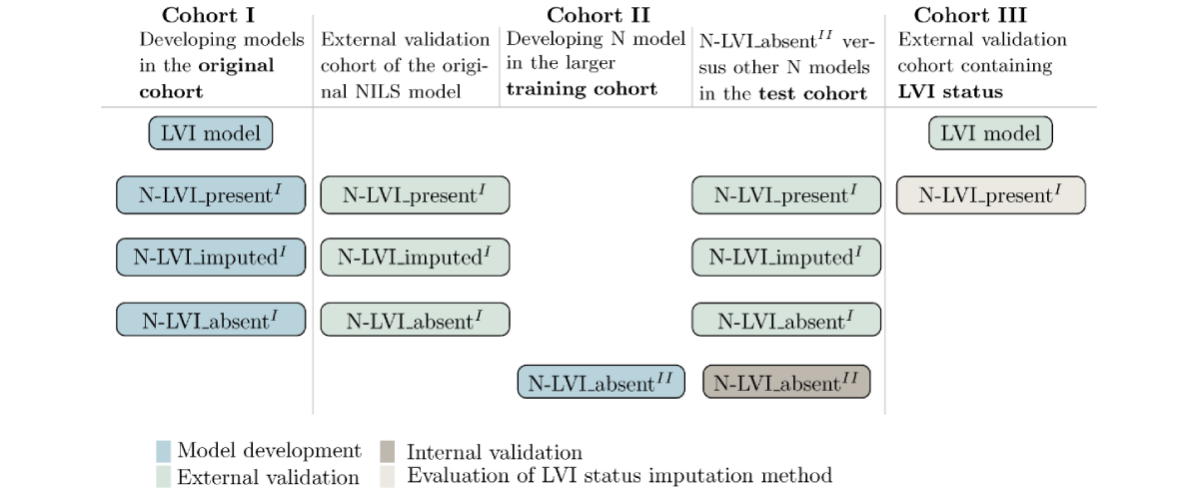
Models developed and evaluated in the study. Three nodal (N) models were developed to account for the lack of data on lymphovascular invasion (LVI) status in cohort II. The external validation was made in cohort II (Swedish National Quality Register for Breast Cancer; n=18,633). A new N model was developed in the training cohort (n=14,906) of cohort II, and its performance was compared with that of the 3 other N models in the test cohort (n=3727) of cohort II. An LVI model was developed to predict the LVI status for patients without documented LVI status in cohort I, and the LVI model was externally validated in cohort III. In addition, different alternatives for LVI imputation were tested in the model N-LVI_present^I^ in cohort III.

This was an observational diagnostic study. Because of the absence of information on LVI status in cohort II, a total of 3 N models trained in cohort I (N-LVI_present^I^, N-LVI_imputed^I^, and N-LVI_absent^I^; Figure 2) were developed to externally validate the original NILS model [7]. Each of the 3 models had different access to values for the LVI status. When applicable, missing data on the LVI status were imputed using an LVI model (LVI model in Figure 2). The model N-LVI_present^I^ was developed using only patients with a documented LVI status (613/761, 80.6% patients in cohort I). For the model N-LVI_imputed^I^, patients with missing values for LVI status (148/761, 19.4% patients) had these predicted using the LVI model, and the model was trained on all 761 patients in cohort I. The model N-LVI_absent^I^ was developed without access to LVI status in all 761 patients in cohort I. The LVI model was developed based on 613 patients in cohort I with a documented LVI status.

The 3 available cohorts enabled us to externally validate the original NILS model [7] and investigate the effect of imputed LVI status values on N model predictions. Imputations by the LVI model were further evaluated in the model N-LVI_present^I^ (refer to the LVI Model Evaluation section). The considerably larger size of cohort II also enabled the development of a new N model (N-LVI_absent^II^; Figure 2) in a large population-based cohort.

Cohort II was categorized into a training and a test data set of 80%/20% (14,906/3727) stratified by N status to compare the performance of the model N-LVI_absent^II^ with that of N models N-LVI_present^I^, N-LVI_imputed^I^, and N-LVI_absent^I^. The model N-LVI_absent^II^ was developed using the training data set whereas the test data set was set aside for comparison with the other developed N models.

### Model Development and Selection

The process of training the LVI model and the 4 N models was similar to that by Dihge et al [7] but with minor modifications owing to different access to data, as presented in the Study Population section, an ensemble MLP was developed for each examined hyperparameter combination, and every network in the ensemble was trained using 5-fold cross-validation, stratified by the outcome distribution. The mean validation AUC of each ensemble was compared to identify the hyperparameter combination that yielded the highest validation AUC value. One difference from the original model development was the use of random search instead of grid search, where each learning algorithm was assigned randomly selected hyperparameters, given a range of values. This hyperparameter optimization method is more efficient than iterating over all possible hyperparameter combinations [31].

### Missing Data

The 3 cohorts had between 1% and 2% missing values and 72% to 90% complete-case patients (Multimedia Appendices 3-5). Missing LVI status was assumed to be missing at random conditional on the other predictors, and other values were assumed to be missing completely at random. In the original NILS model, missing data were handled using multiple random imputation. In this study, missing data were imputed either by multiple random imputation or by the LVI model. Although the methodology used to develop the LVI model can be applied to other variables with missing data, it was decided to be relevant only for LVI because of its importance for the N prediction models.

All cases with missing LVI status values were predicted using the LVI model. During the development of the model N-LVI_imputed^I^, the LVI model was used to predict the LVI status of 148 patients lacking information on LVI status in cohort I at the beginning of each fold in the 5-fold cross-validation. For each training epoch, the LVI status was set to positive or negative, given the probability of the prediction. Missing values among other variables were imputed using multiple random imputation, where a missing value was randomly replaced by a value in the present data distribution for the corresponding variable. This procedure was repeated at the beginning of each training epoch.

### LVI Model Evaluation

To evaluate the LVI model developed in cohort I, a total of 3 types of imputations of LVI status were compared with the original values for LVI status in cohort III. The comparison was made using the N status predicted by the N-LVI_present^I^ model. Subsequently, the three types of imputation were (1) the probability predicted using the LVI model; (2) the corresponding category (LVI positive or LVI negative) given the probability of the prediction; and (3) the corresponding category of the prediction given a cutoff of 0.3, matching the distribution of the LVI predictions in the internal cross-validation with that of the development cohort.

The imputation option yielding the highest validation AUC for N status, calculated as the mean of the N-LVI_present^I^ model’s predictions over 25 imputed data sets, was chosen for the imputation of the LVI status in cohort II. Calibration curves of the observed versus mean predicted probabilities were used to visualize the LVI model calibration.

### N Model Evaluation

The N model validation AUC was calculated as the mean of the AUCs over 25 data sets imputed for missing values, and the LVI status was imputed by the LVI prediction model for each data set when applicable. In addition, a secondary outcome for the N models was the proportion of patients that could be omitted from SLNB while maintaining the FNR at 10% (the generally accepted FNR of SLNB [25]). The successful criteria for developing an N model to identify potential candidates for omitting SLNB in every fifth patient with cN0 breast cancer were established in advance.

Model predictions were recalibrated to the prevalence in the external validation cohort to account for the different N status distributions of cohorts I and II [32]. In addition, calibration curves of the observed versus mean predicted probabilities were used to visualize the model calibration. Finally, decision curves [33] were analyzed to examine the standardized clinical benefit [34] of the N models, where the threshold probabilities were set to the range of the acceptable level for the FNR (0%-10%).

### Software and Hardware

All parts of the study were conducted using Python (version 3.9.7; Python Software Foundation) [35] and TensorFlow (version 2.6.0; Google Brain Team) [36], with a computer equipped with an Intel Core i7-8700K CPU at 3.70 GHz and 2 GeForce RTX 2080 GPUs.

### Ethical Considerations

The Regional Ethics Committee at Lund University, Sweden, approved cohort I for the study (LU 2013/340), and ethics approval was obtained for the use of an opt-out methodology. Cohorts II and III received approval from the Swedish Ethical Review Authority under reference numbers 2019-02139 and 2021-00174, respectively, for the study. Written informed consent for participation was not required for this noninterventional study in accordance with the national legislation and institutional requirements. All patients included in the study were given the option to opt out. The data sets generated and analyzed from anonymized data were separated from personal identifiers. Data are not publicly available because of privacy and ethical restrictions, and information is not made available or disclosed to unauthorized individuals, entities, or processes.

## Results

### Study Population and Data Availability

Access to variables differed between the large population-based register (cohort II; Table 1) and the data obtained from medical records in cohorts I and III (Multimedia Appendices 6 and 7, respectively). BMI and tumor localization data were not routinely registered in the NKBC, and these 2 variables were excluded.

**Table 1:** Patient and tumor characteristics for cohort II (n=18,633).

		All patients	Node negative (n=14,829)	Node positive (n=3804)
Age (years), median (range)		65 (22-95)	65 (22-95)	63 (23-94)
**Menopausal status, n (%)**				
	Premenopausal	3336 (19)	2515 (18.04)	821 (22.9)
	Postmenopausal	14,224 (81)	11,457 (81.96)	2767 (77.12)
	Missing^a^	1073 (5.76)	857 (5.8)	216 (5.7)
**Mode of detection, n (%)**				
	Mammographic screening	10,816 (58.17)	8992 (60.78)	1824 (48.01)
	Symptomatic presentation	7777 (41.83)	5802 (39.22)	1975 (51.99)
	Missing	40 (0)	35 (0)	5 (0)
Tumor size (mm), median (range)		15 (1-50)	14 (1-50)	19 (1-50)
**Multifocality, n (%)**				
	Absent	15,537 (83.54)	12,730 (85.98)	2807 (74)
	Present	3061 (16.46)	2075 (14.02)	986 (26)
	Missing	35 (0)	24 (0)	11 (0)
**Histological type, n (%)**				
	No special type	14,322 (76.86)	11,325 (76.37)	2997 (78.79)
	Lobular	2387 (12.81)	1862 (12.56)	525 (13.8)
	Other invasive, including mixed types	1924 (10.33)	1642 (11.07)	282 (7.4)
**Nottingham histological grade, n (%)**				
	1	4112 (22.32)	3600 (24.57)	512 (13.6)
	2	9672 (52.49)	7595 (51.83)	2077 (55.03)
	3	4643 (25.2)	3458 (23.6)	1185 (31.4)
	Missing	206 (1.1)	176 (1.2)	30 (1)
**Estrogen receptor status, n (%)**				
	Negative (<1%)	1490 (8.32)	1199 (8.41)	291 (7.9)
	Positive (≥1%)	16,423 (91.68)	13,053 (91.59)	3370 (92.05)
	Missing	720 (3.9)	577 (3.9)	143 (3.8)
**Progesterone receptor status, n (%)**				
	Negative (<1%)	2672 (15.14)	2182 (15.56)	490 (13.5)
	Positive (≥1%)	14,973 (84.86)	11,839 (84.44)	3134 (86.48)
	Missing	988 (5.3)	808 (5.4)	180 (4.7)
**Human epidermal growth factor receptor 2 status, n (%)**				
	Negative	16,288 (88.67)	12,989 (88.87)	3299 (87.88)
	Positive	2082 (11.33)	1627 (11.13)	455 (12.1)
	Missing	263 (1.4)	213 (1.4)	50 (1)
**Ki67 (%)**				
	Values, median (range)	20 (0-100)	20 (0-100)	24 (1-100)
	Missing, n (%)	133 (0.7)	123 (0.8)	10 (0)

^a^The number of missing values is shown for noncomplete case variables.

### LVI and N Model Evaluation

#### Training and Validation of the LVI Model

**Figure 3:**
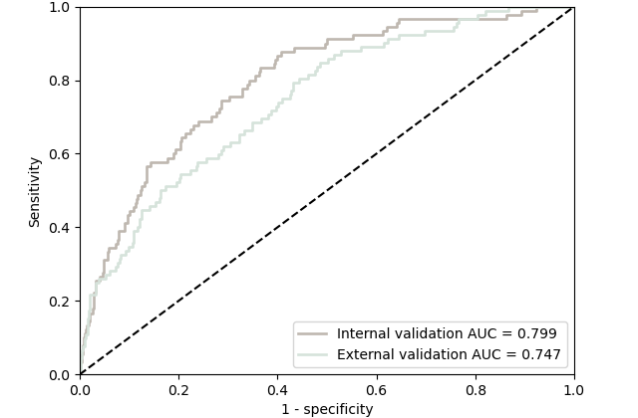
ROC curve for the LVI model. The lymphovascular invasion model had a discriminatory performance area under the receiver operating characteristic curve (AUC) of 0.799 (95% CI 0.751-0.864) in the internal validation and an AUC of 0.747 (95% CI 0.694-0.799) in the external validation. ROC: receiver operating characteristic.

The LVI model was trained on 613 patients in cohort I and evaluated in the validation part of cohort III (n=525; Figure 2; Multimedia Appendix 7). The model had an internal cross-validation AUC of 0.799 (95% CI 0.751-0.846) and an external validation AUC of 0.747 (95% CI 0.694-0.799; Figure 3). In addition, the LVI model showed good calibration in external validation (Multimedia Appendix 8). The final architecture for the LVI and N models can be found in Multimedia Appendix 9.

All alternatives for LVI imputation were evaluated in cohort III using the N model N-LVI_present^I^. The model N-LVI_present^I^ imputed with probabilistically drawn categorical values of LVI status performed slightly better than the other options (Table 2); therefore, this type of LVI imputation was subsequently used.

**Table 2:** Area under the receiver operating characteristic curve (AUC) for the nodal status predictions of the model N-LVI_present^I^ for different strategies for imputing values of lymphovascular invasion (LVI) status. The highest AUC, except when using the original LVI values, was obtained when imputing LVI status using the probabilistic imputation, this is why we chose to use this method for LVI imputation in the subsequent analysis.

	Original LVI status	LVI status imputed by the predicted probability	LVI status imputed by probabilistically categorical imputation	LVI status imputed by categorical imputation with threshold 0.3
N-LVI_present^I^, AUC (95% CI)	0.750 (0.704-0.795)	0.737 (0.689-0.783)	0.740 (0.693-0.784)	0.738 (0.691-0.783)

#### External Validation of the Original NILS Model

**Figure 4:**
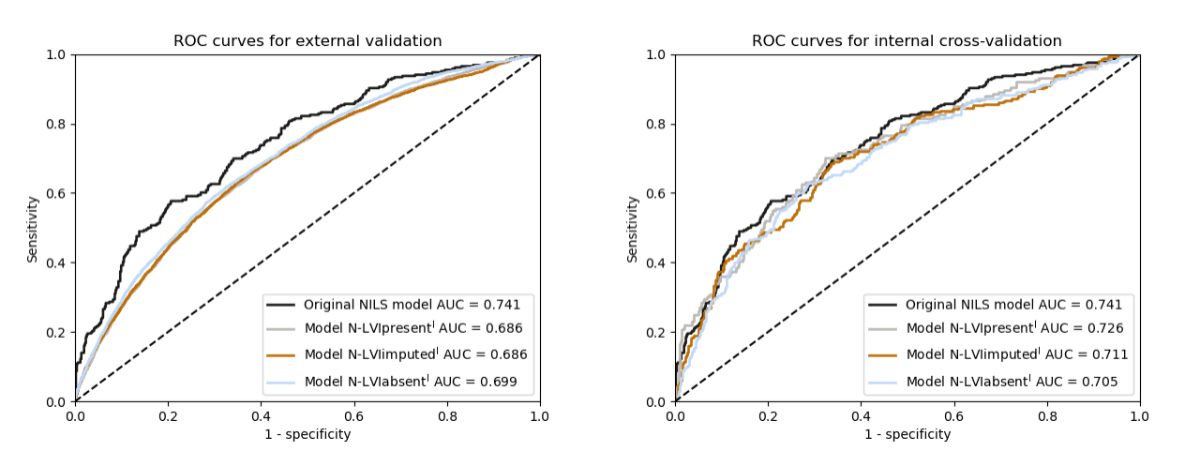
The receiver operating characteristic (ROC) curve for the external validation (left) and the internal validation (right) of the original noninvasive lymph node staging (NILS) model in the study by Dihge et al [7]. The models of this study had access to slightly different variables and a different number of patients in the training cohort than that of the original NILS model. The models N-LVI_present^I^ and N-LVI_imputedI both included LVI status, whereas N-LVI_absentI did not. Note that the original model was cross-validated with area under the receiver operating characteristic curve (AUC) 0.740 in the study by Dihge et al [7], which was an average of 5 runs. The ROC curve of the original NILS model is in this figure represented by the run closest to the mean; AUC 0.741.

To externally validate the original NILS model in cohort II without information on the LVI status, 3 N models (N-LVI_present^I^, N-LVI_imputed^I^, and N-LVI_absent^I^) were developed for cohort I (n=761), as shown in Figure 2. The original NILS model was internally cross-validated, with an AUC of 0.740 (95% CI 0.723-0.758) [7]. In the external validation in cohort II (n=18,633), both the N-LVIpresent^I^ and N-LVIimputed^I^ models reached an AUC of 0.686 (95% CI 0.677-0.695; Figure 4, left [7]). Furthermore, upon validation, the model N-LVIabsent^I^ reached an AUC of 0.699 (95% CI 0.690-0.708). The classification performance of all N models is summarized in Multimedia Appendix 10.

#### The Impact of the LVI Model on the Overall N Status Predictions

The internal validation of the N models showed a higher performance for models N-LVI_present^I^ and N-LVI_imputed^I^ using LVI status (AUC 0.726, 95% CI 0.681-0.768 and AUC 0.711, 95% CI 0.762-0.750, respectively), compared with that of model N-LVI_absent^I^ not including the LVI status (AUC 0.705, 95% CI 0.665-0.744; Figure 4, right). For external evaluation of the models N-LVI_present^I^ and N-LVI_imputed^I^, the LVI model was used to predict the LVI status in cohort II.

When externally validated in cohort II (n=18,633), the models N-LVI_present^I^, N-LVI_imputed^I^, and N-LVI_absent^I^ showed similar performances (Figure 4, left). Therefore, the rest of the external validation focused on the model developed without access to the LVI status, N-LVI_absent^I^. In the calibration plot, the model N-LVI_absent^I^ demonstrated slightly lower predictions than the true values in the external validation (Multimedia Appendix 11). However, when transforming the predictions in relation to the prevalence of N0 in the validation cohort, the calibration of the model N-LVI_absent^I^ was satisfactory.

#### Comparison Between Developed N Models

**Figure 5:**
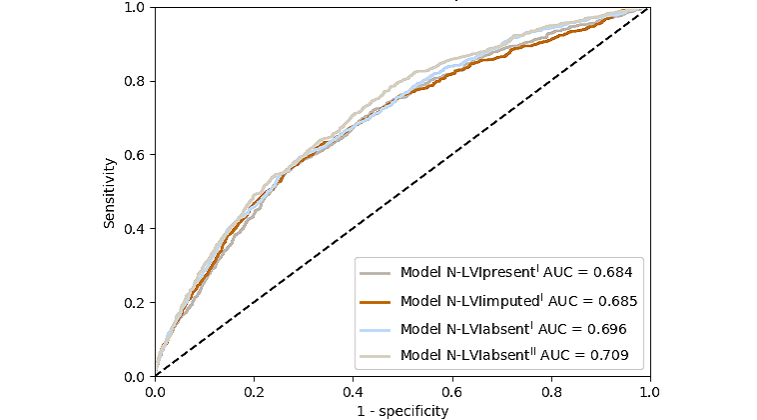
ROC curves for the developed N models. Validation in the test cohort (n=3727) of cohort II for the nodal (N) models N-LVI_present^I^, N-LVI_imputed^I^, N-LVI_absent^I^, and the new N model N-LVI_absent^II^ developed in a larger cohort. AUC: area under the receiver operating characteristic curve; ROC: receiver operating characteristic.

The fourth N status model, N-LVI_absent^II^, was developed in NKBC, a large population-based cohort. The cohort was considerably larger (training cohort: 14,906/18,663, 80%) than the development cohort for the other 3 N models and the original NILS model [7] (cohort I). The test cohort of cohort II (3727/18,663, 20%), set aside before the development of model N-LVI_absent^II^, was used to compare the performance of the developed N models. The models N-LVI_present^I^, N-LVI_imputed^I^, and N-LVI_absent^I^ reached AUC of 0.684 (95% CI 0.663-0.705), 0.685 (95% CI 0.663-0.706), and 0.696 (95% CI 0.676-0.717), respectively (Figure 5). The model N-LVI_absent^II^ reached a slightly higher AUC of 0.709 (95% CI 0.688-0.729). The calibration plot for the model N-LVI_absent^II^ is shown in Multimedia Appendix 12.

#### Assessments of Potential Clinical Utility of the N Models

**Figure 6:**
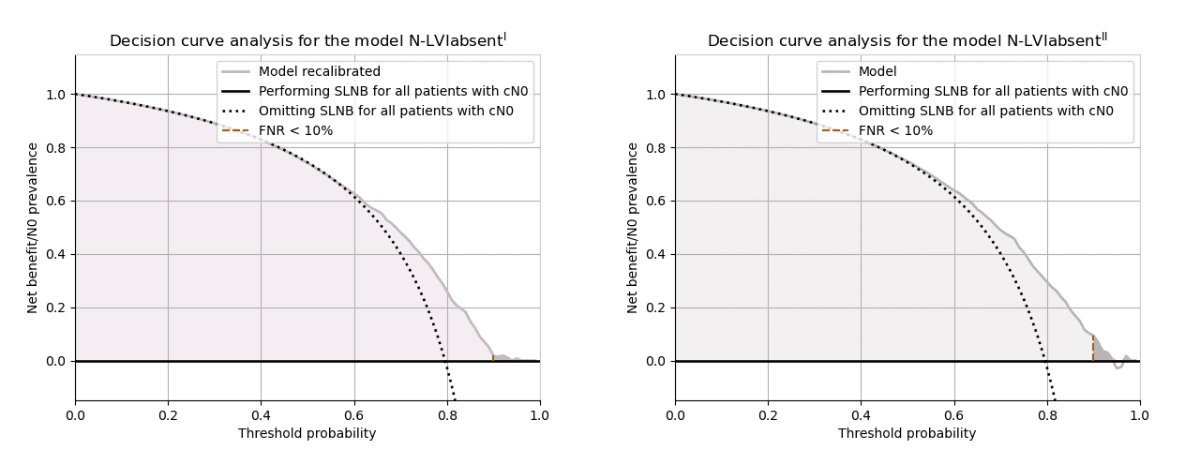
Decision curves showing the standardized net benefit of the model N-LVI_absent^I^ (recalibrated; left) and the model N-LVI_absent^II^ (right). The black horizontal line represents the scenario of all patients being diagnosed as node negative; hence, no sentinel lymph node biopsy (SLNB) is performed. The colored function represents the diagnosis by the model. The golden, vertical (dashed) line at a threshold of approximately 0.9, separating the lighter color from the darker, shows the threshold for false-negative rate (FNR) <10%. When all patients are considered node positive and diagnosed through SLNB, the standardized net benefit is, by definition, 0. Notably, the darker, colored area does not represent the patients spared from surgery. Rather, it displays the standardized net benefit of the model where FNR<10%. cN0: clinically node negative.

External validation of the N models before recalibration showed potential in sparing approximately 20% of patients with cN0 breast cancer from axillary surgery when using an FNR of <10%. When recalibrating the predictions for the model N-LVI_absent^I^, the number decreased to approximately 16%). However, the new N model N-LVI_absent^II^ developed in cohort II could potentially spare 24% of the patients with cN0 breast cancer from SLNB. The standardized decision curve analyses (Figure 6) specifically showed the range of predictions where patients could benefit from using the 2 prediction models. The standardized decision curve analysis for the original predictions of N-LVI_absent^I^ before recalibration is presented in Multimedia Appendix 13.

## Discussion

### Principal Findings

The proportion of patients diagnosed with early-stage breast cancer is increasing [4]. Along with improvements in adjuvant therapy, surgical treatment is becoming more conservative. Most patients with early-stage breast cancer have benign lymph nodes and would benefit from preoperative noninvasive staging of the axilla [3,4]. In this study, we externally validated a previously published N model [7] in a national, large, population-based register cohort (n=18,633) without access to the LVI status and developed a new N model within the same cohort. Notably, the discriminatory performance (AUC 0.709, 95% CI 0.688-0.729) of the new N model (N-LVI_absent^II^) developed in the large population-based cohort demonstrated that routine clinicopathological register data can be used to develop an N model to identify 24% of patients with cN0 for whom surgical axillary staging could be circumvented. The model developed in cohort I without access to data on LVI status (N-LVI_absent^I^) achieved an AUC of 0.699 (95% CI 0.690-0.708) and the potential to omit 16% of patients Malfrom SLNB. The use of fewer variables and, in some cases, fewer patients was expected to result in a slight decrease in the performance of the models in this study compared with that of the original model. The study is conducted in accordance with the TRIPOD statement as displayed in Multimedia Appendix 14.

### Comparison With Prior Studies

Multiple studies have investigated the discriminatory performance of nomograms in predicting the N status using retrospective clinicopathological data alone or in combination with imaging features [6,8-17]. We aimed to externally validate and further develop a diagnostic tool for the noninvasive staging of N status using only routinely available clinicopathological data that can be captured in the preoperative setting to improve the clinical utility of the model. Li et al [8] and Gao et al [12] developed nomograms using solely clinicopathological data that can be obtained preoperatively. However, these studies did not specify whether the data were extracted from the preoperative or postoperative setting. Li et al [8] had the advantage of a very large cohort (n=184,532); unfortunately, combining external validation data with parts of the development cohort resulted in an inaccurate external validation (AUC 0.718). Gao et al [12] developed a nomogram based on 6314 patients with external validation on 503 patients, where the shift from training and internal validation to external validation increased from an AUC of 0.715 and 0.688 to an AUC of 0.876, respectively. This large discriminatory increase in external validation is unexpected and warrants questioning the validity of the model.

One possibility for the transition from postoperative to preoperative variables is the use of imaging features. Mao et al [15] developed a nomogram using CESM-reported lymph node status and a radiomics signature to predict axillary lymph node status. In addition, the nomogram was externally validated on only 62 patients with an AUC of 0.79 (95% CI 0.63-0.94). Using only features that can be obtained preoperatively is an advantage in the study by Mao et al [15]. However, additional larger external validation is required to confirm the results of the study. Furthermore, CESM is not part of the mammography screening program or routine workup for suspected breast malignancies, limiting the clinical feasibility of the study. Bove et al [5] developed a support vector machine (SVM) classifier for clinical data and a SVM for radiomics data to predict N status. They used soft voting, which combines the probabilities of each prediction in the 2 models. They chose the prediction with the highest total probability, which resulted in an AUC of 0.886 on the holdout test set. Combining pre- and postoperative variables is a limitation of the study, and the axillary US is an operator-dependent imaging modality. However, the results show the potential for using imaging features in machine learning models for the noninvasive staging of N status. The SVM classifier had an AUC of 0.739 using only postoperative clinicopathological data, similar to that of the original NILS model [7]. However, both the training (n=114) and test (n=28) data sets were small; therefore, a larger external validation is needed to confirm the results.

In this study, the LVI model, trained using only routine clinicopathological variables and developed to increase the feasibility of the NILS models in the preoperative setting, had an external validation AUC of 0.747 (95% CI 0.694-0.799). To the best of our knowledge, this is the first LVI model to be incorporated into an N model. Preoperative assessment of LVI on CNB is challenging, and several models have been developed to predict the LVI status. For example, Shen et al [37] developed a logistic regression model for the LVI status using clinicopathological variables (n=392). Although the model reached an AUC of 0.670 (95% CI 0.607-0.734) in the training data set, it was not validated further. In addition, others have investigated the importance of radiomics features for predicting LVI status, for example, digital mammography features [38] with LVI prediction specificity of 98.8% in the development cohort and MRI features [39] with an AUC of 0.732 in the test data set. However, while highlighting the potential for predicting LVI status using radiomics, the data used are not part of the diagnostic workup for breast cancer, thus limiting clinical feasibility.

Despite the AUC of 0.747 for the LVI model in this study, the imputation of values for the LVI status did not improve the discriminatory performance of the N models in the large population-based register cohort (NKBC). One problem with developing prediction models for the classification question at hand is the scarcity of larger cohorts including relevant clinicopathological data such as LVI as well as the lack of identified strong predictors of LVI. Those reported in the literature include tumor size, HER2 status, and Ki67 [37] and were included in the LVI model as well as in the N model, which might have hampered the signal to predict N status by imputing LVI. Novel approaches using image analysis seem to capture features with superior discriminatory capacity [38,39]. Moreover, the reliability and distribution of data, such as multifocality, may change in the preoperative setting [40], which could change the prerequisites for predicting LVI status. Given the growing evidence on the significance of LVI status as a predictor of axillary N status [7,11,22,41], further evaluation of the presented LVI model is warranted.

### Potential Clinical Utility

Omitting SLNB in subgroups of patients is consistent with the American Society of Clinical Oncology guidelines from 2021 [2], stating that SLNB is optional for all patients aged ≥70 years with cN0, ER+, and HER2− if the patient received adjuvant endocrine therapy. In this study, using only routine clinicopathological data, the models developed without access to LVI status in cohort I (recalibrated) and cohort II presented the potential to spare 16% to 24% of patients with cN0 from SLNB, irrespective of age and tumor subtype. Providing clinicians and patients with a decision support tool enabling the identification of one quarter of patients as eligible for abstaining SLNB could enhance the adoption of the 2021 American Society of Clinical Oncology guidelines [2]. In addition, a health economic study concluded that the NILS model is cost-effective [42]. If lymphedema is considered to negatively affect patients’ quality of life, the NILS model also showed a net health gain [42].

### Strengths and Limitations

Criticism has been raised against the use of small sample sizes in the development and external validation of machine learning models in oncology as well as the poor handling of missing data [39]. Accordingly, we aimed to externally validate the original NILS model [7] in a nationwide and large population-based register cohort (n=18,633) and to develop a new NILS model within this larger cohort (14,906/18,633, 80%). Using a large population-based register cohort is advantageous in the following two ways: (1) its consecutive nature constitutes a good approximation of the true distributions of the population and (2) it demonstrates the reality of data handling where input data will comprise missing values and occasional mistakes in documentation. The limitations of using quality registry data are the risk of misclassification and missing data, which were handled by meticulous data curation and exclusion of patients without properly defined or missing variables (1091/23,264, 4.69%). Moreover, the register lacks information on race; hence, the generalizability to other populations outside Sweden has to be proven in external data sets. Importantly, our findings demonstrate that register data can be used to create an N model with results just as satisfactory as those obtained from more meticulously curated data, including the LVI status. Our external validation of the original NILS model [7] was performed in a temporally, geographically, and domain-wise different cohort from the original development cohort. We presented calibration and net benefit curves to demonstrate the utility of the models. In addition, the 1091 patients in cohort II with missing or incongruent data for axillary surgery and lymph node status (Figure 1) showed a similar distribution of clinical variables (data not shown) as the final study population of cohort II. Therefore, there was no indication of selection bias.

Another strength of our study is the thorough management of missing data using both the LVI model and multiple random imputation. Our comprehensive handling of missing values may increase the utility of N models in a clinical preoperative setting. It also showed that for the discriminatory performance in N staging, the manner in which the predictions of LVI status were presented to an N model was of minor importance. However, this requires further investigation in the preoperative setting and use of an LVI model with an even higher discriminatory performance to completely rule out the potential advantage of MLP LVI predictions in NILS.

However, this study had some limitations. First, the models were developed using a combination of variables available before and after surgery to externally validate the original NILS model [7], which is based on preoperative and postoperative variables. Further development of the NILS concept is an ongoing validation of the NILS model using exclusively preoperative variables [29]. Second, the generalizability of the LVI and N models developed in cohort I can be affected by the smaller size of the development cohorts, which can be considered a weakness of the study. Therefore, regularization of the networks and 5-fold cross-validation were used to minimize overfitting. The drop in performance from the internal to external validation was small for all models, which is a clear strength of our findings.

Recalibration was performed for the model developed without access to LVI status in cohort I (N-LVI_absent^I^) because of the different prevalence of benign lymph nodes in cohorts I and II (497/761, 65.3% pathological benign nodal status [pN0] vs 14,829/18,633, 79.58% pN0). No recalibration was performed for the LVI model because the prevalence of a positive LVI status was similar in cohorts I and III. Notably, when transforming the N status predictions in relation to the new prevalence, the calibration and the overall net benefit of the model N-LVI_absent^I^ improved, whereas the fraction of patients to be spared from SLNB decreased. Therefore, to potentially increase the number of candidate patients to be omitted from SLNB, an important future development of the model could be to evaluate it using partial AUC [43] or concordant partial AUC [44]. The model selection is then based on the model’s performance under specific conditions, for example, FNR of <10%, which could optimize the model performance for patients most likely to benefit from the prediction. Another option is to investigate the modification of the loss function when training the MLP to optimize the algorithm for the largest number of patients to be omitted from SLNB while maintaining the FNR of <10%.

An additional strength of this study was the use of 3 disjoint cohorts for model development and validation. After model development, 2 patients in cohort I were incorrectly classified as N0 instead of N+. However, these 2 patients corresponded to <1% of the cohort and did not affect the overall results. Cohort II demonstrated high validity and a high coverage of key variables [28]. An independent researcher validated and monitored cohort III according to a specific quality assurance protocol to ensure well-characterized data. All variables, except 1, were defined in coherence; the mixed histological type was categorized as missing in cohort III. However, this should have a limited effect on the results because mixed histological type is rare (approximately 5%) [45]. To avoid potential dependency and information leakage between the 2 tumors and N statuses, we excluded patients with bilateral tumors. The exclusion limits the target group to a minor extent, as bilateral cancers are generally diagnosed in <5% of patients [46].

### Future Studies

Future steps include a prospective external validation of the NILS concept in a larger cohort and an evaluation of the incorporation of LVI predictions in a NILS model in the preoperative setting. External validation of the LVI model in a Norwegian breast cancer cohort is also planned. The feasibility of using register data for prediction modeling demonstrates the possibility of using larger and less-curated databases in machine learning models for NILS.

Implementing neural network models that are equal to or superior to linear models allows extending the model to more complex data that cannot be handled by logistic regression in end-to-end learning. This enables less human interference, simpler implementation, and models to optimize the entire task. Therefore, to potentially improve the discriminatory performance of noninvasive staging of lymph nodes for future clinical implementation, additional types of data conferring to the knowledge of lymphatic spread should ideally be investigated. Imaging features are both preoperatively available and have shown high discriminatory performance in N prediction models [5,9-11,13-15]. The possibility of incorporating mammography-based radiomics for the preoperative prediction of N status is intriguing. However, there are challenges in techniques to improve segmentation efficiency and reduce subjective inconsistency from manual segmentation for intratumoral and peritumoral feature extraction. In addition, molecular subtypes are associated with the outcome as well as N status, and the difficult-to-treat triple-negative subtype has the lowest risk of N metastasis compared with luminal tumors [6]. Consequently, models based on gene expression analysis have shown potential in correctly identifying patients with N0 status in specific subtypes of breast cancer, such as luminal A [47], ER+/HER2− [48], and triple-negative tumors [49], to capture additional aspects of lymphatic spread, such as immune signatures. Gene expression data have also shown the potential to increase the number of candidate patients to be omitted from SLNB when combined with clinicopathological data compared with predicting N status using clinicopathological data alone [50]. The added cost and effort of gene expression analysis should be considered in relation to avoiding SLNB. In contrast, gene expression–based assays, especially RNA sequencing, also have the potential to provide additional information through prognostic or predictive signatures. Therefore, planned extensions of the NILS model include mammography images and gene expression data, mainly focusing on molecular subtypes and immune signatures.

### Conclusions

We externally validated the original NILS model [7] in a large population-based register cohort, with a discriminatory performance of 0.699 (95% CI 0.690-0.708). Prediction of LVI status did not improve the performance of the N model, despite its documented importance in the prediction of axillary stage. A new MLP model for predicting N status was developed in a large population-based register cohort, demonstrating the feasibility of developing a prediction model for noninvasive N staging using register data comprising only variables available in the preoperative setting and, notably, no information on LVI status (AUC 0.709, 95% CI 0.688-0.729). Therefore, future studies should evaluate the LVI model in the preoperative setting, the ongoing preoperative validation of the NILS concept, and extend the NILS model with preoperative and routinely available data such as mammography images and gene expression data.

## Appendix

Multimedia Appendix 1 Patient selection for cohort I.

Multimedia Appendix 2 Patient selection for cohort III.

Multimedia Appendix 3 Data characteristics of the development cohorts for nodal status models.

Multimedia Appendix 4 Data characteristics of the validation cohorts for nodal status models.

Multimedia Appendix 5 Data characteristics of the development and evaluation cohorts for the lymphovascular invasion status model.

Multimedia Appendix 6 Patient and tumor characteristics in cohort I.

Multimedia Appendix 7 Patient and tumor characteristics in cohort III.

Multimedia Appendix 8 Calibration of the lymphovascular invasion model in cohort III.

Multimedia Appendix 9 Model architecture of the lymphovascular invasion and nodal models.

Multimedia Appendix 10 Sensitivity, specificity, and false-negative rate of nodal models.

Multimedia Appendix 11 Calibration and recalibration of the nodal status model N-LVI_absent^I^ in the test cohort of cohort II.

Multimedia Appendix 12 Calibration of the N status model N-LVI_absent^II^ in the test cohort of cohort II.

Multimedia Appendix 13 Standardized decision curve analysis for the original predictions of the model N-LVI_absent^I^.

Multimedia Appendix 14 Transparent Reporting of a multivariate prediction model of Individual Prognosis Or Diagnosis (TRIPOD) checklist.

## Abbreviations

AUC: area under the receiver operating characteristic curve
CESM: contrast-enhanced spectral mammography
cN0: clinically node-negative
CNB: core needle biopsy
ER: estrogen receptor
FNR: false-negative rate
HER2: human epidermal growth factor receptor 2
LVI: lymphovascular invasion
MLP: multilayer perceptron
MRI: magnetic resonance imaging
N: nodal
NHG: Nottingham histological grade
NILS: noninvasive lymph node staging
NKBC: Swedish National Quality Register for Breast Cancer
pN0: pathological benign nodal status
PR: progesterone receptor
SLNB: sentinel lymph node biopsy
SVM: support vector machine
TRIPOD: Transparent Reporting of a multivariate prediction model of Individual Prognosis Or Diagnosis
US: ultrasound

## Data Availability

The raw data sets are available only upon reasonable request because of privacy and ethical restrictions. The data are not publicly available because of these restrictions. The code is available upon reasonable request.
