# Supplementary figures and images for: Noninvasive Staging of Lymph Node Status in Breast Cancer Using Machine Learning: External Validation and Further Model Development
Source: JMIR Cancer. 2023 Nov 20;9:e46474. doi: 10.2196/46474 (PMC10696498; doi:10.2196/46474)

**Figure S1. Patient selection for Cohort I.**

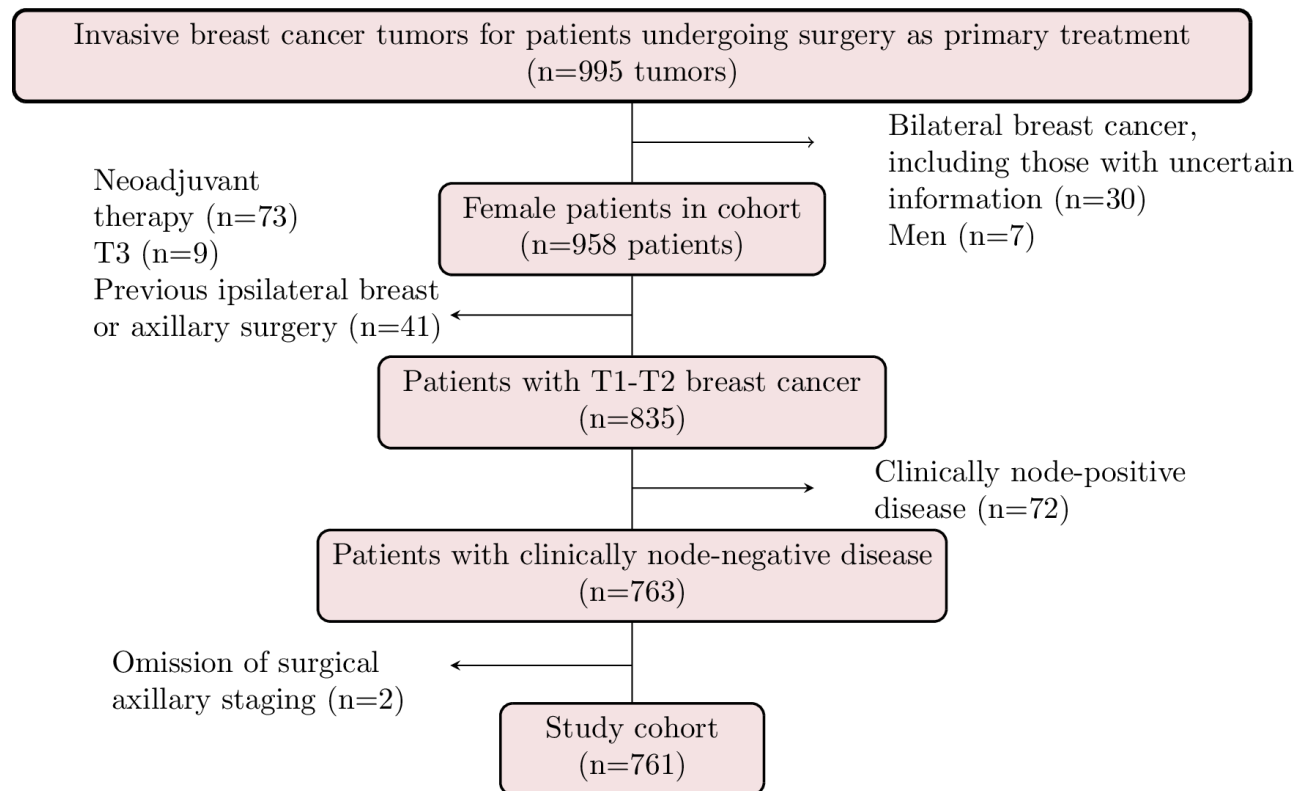

Supplement: Multimedia Appendix 1 [file cancer_v9i1e46474_app1.pdf]

*Figure S3. Calibration of the LVI model in Cohort III.*

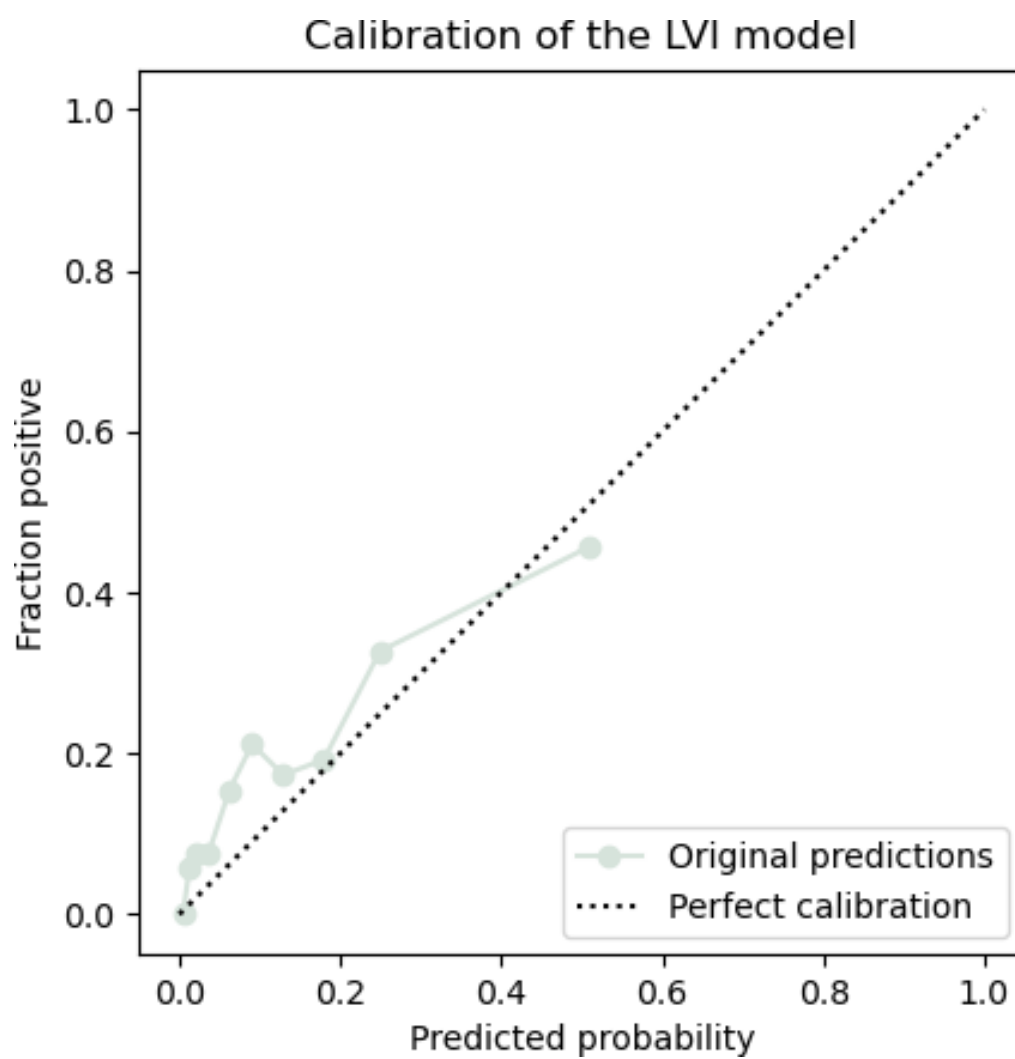

Abbreviation:

LVI, lymphovascular invasion

Supplement: Multimedia Appendix 8 [file cancer_v9i1e46474_app8.pdf]
