# Supplementary material for: Noninvasive Staging of Lymph Node Status in Breast Cancer Using Machine Learning: External Validation and Further Model Development
Source: JMIR Cancer. 2023 Nov 20;9:e46474. doi: 10.2196/46474 (PMC10696498; doi:10.2196/46474)
Supplement: Multimedia Appendix 2 [file cancer_v9i1e46474_app2.pdf]

Figure S2. Patient selection for Cohort III.

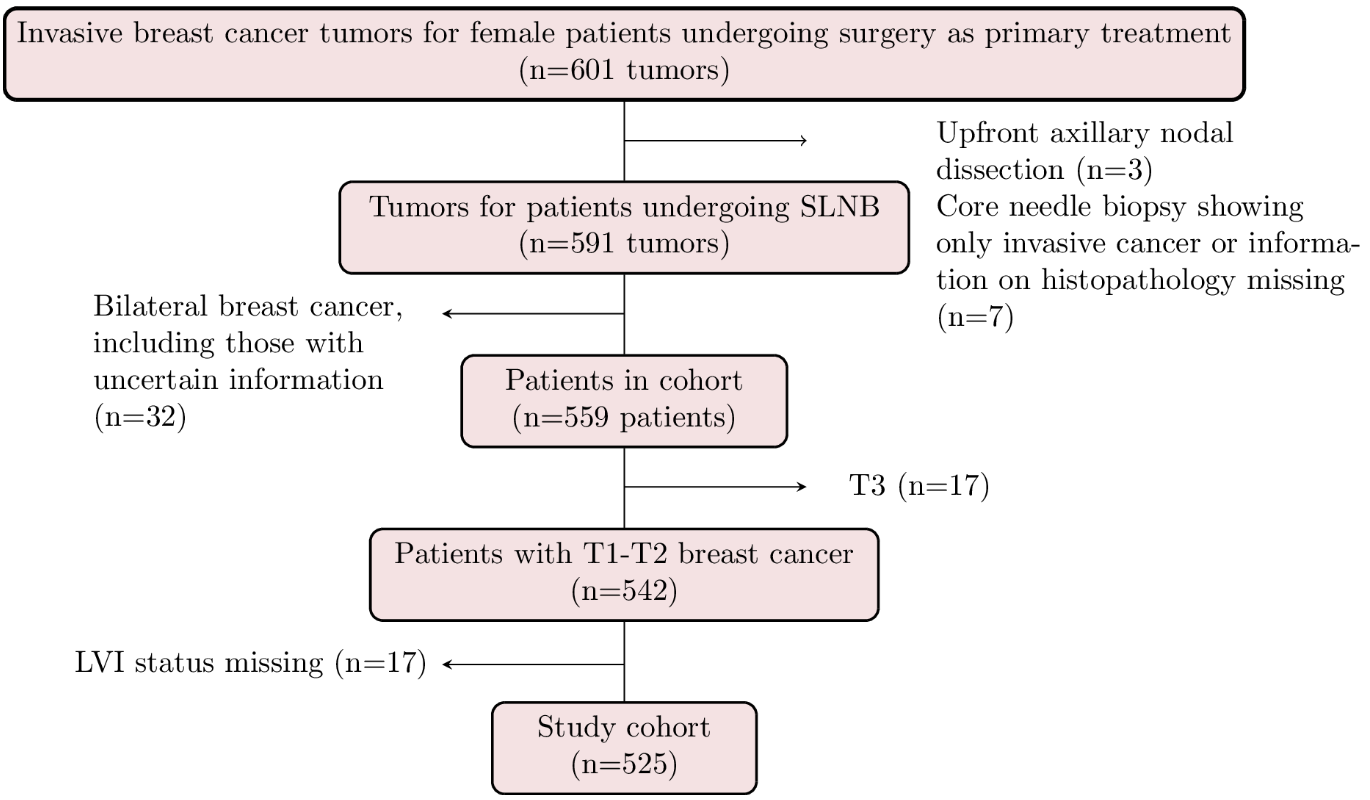

Abbreviations:

SLNB, sentinel lymph node biopsy

LVI, lymphovascular invasion
