# Supplementary material for: Noninvasive Staging of Lymph Node Status in Breast Cancer Using Machine Learning: External Validation and Further Model Development
Source: JMIR Cancer. 2023 Nov 20;9:e46474. doi: 10.2196/46474 (PMC10696498; doi:10.2196/46474)
Supplement: Multimedia Appendix 3 [file cancer_v9i1e46474_app3.pdf]

Table S1. Data characteristics in development cohorts for nodal (N) status models. No values were missing for the target variable nodal status.

	N-LVI_present[Ia]	N-LVI_imputed[Ib]	N-LVI_absent[Ic]	N-LVI_absent[IIc]
**Dataset (N)**				
	Cohort I (613)	Cohort I (761)	Cohort I (761)	Training cohort II (14 906)
**Missing values**				
	67 (1%)	157 (2%)	157 (1%)	2814 (2%)
**Complete cases**				
	550 (90%)	550 (72%)	626 (82%)	12 919 (87%)
**N0/N+**				
	413/200 (67%/33%)	497/264 (65%/35%)	497/264 (65%/35%)	11 863/3043 (80%/20%)

[a] Trained on LVI status complete case data.

[b] Trained on data with imputations of missing values of LVI status.

[c] Trained without access to data on LVI status.

Abbreviations:

LVI, lymphovascular invasion

N0, node-negative

N+, node-positive
