# Supplementary material for: Noninvasive Staging of Lymph Node Status in Breast Cancer Using Machine Learning: External Validation and Further Model Development
Source: JMIR Cancer. 2023 Nov 20;9:e46474. doi: 10.2196/46474 (PMC10696498; doi:10.2196/46474)
Supplement: Multimedia Appendix 4 [file cancer_v9i1e46474_app4.pdf]

Table S2. Data characteristics in the validation cohorts for the nodal status models. No values were missing for the target variable nodal status.

	Cohort II (n=18 633)	Test cohort II (n=3727)
**Models**		
	N-LVI_present^{I}<br>N-LVI_imputed^{I}<br>N-LVI_absent^{I}	N-LVI_present^{I}<br>N-LVI_imputed^{I}<br>N-LVI_absent^{I}<br>N-LVI_absent^{II}
**Missing values**		
	3458 (2%)	644 (1%)
**Complete cases**		
	16 207 (87%)	3288 (88%)
**N0/N+**		
	14 829/3804 (80%/20%)	2966/761 (80%/20%)

Abbreviations:

LVI, lymphovascular invasion

N0, node-negative

N+, node-positive
