# Supplementary material for: Noninvasive Staging of Lymph Node Status in Breast Cancer Using Machine Learning: External Validation and Further Model Development
Source: JMIR Cancer. 2023 Nov 20;9:e46474. doi: 10.2196/46474 (PMC10696498; doi:10.2196/46474)
Supplement: Multimedia Appendix 5 [file cancer_v9i1e46474_app5.pdf]

Table S3. Data characteristics in the development and the evaluation cohort, respectively, for the lymphovascular invasion (LVI) status model. No values were missing for the target variable LVI.

	LVI development	LVI evaluation
**Dataset (*n*)**		
	Cohort I (613)	Cohort III (525)
**Missing values**		
	67 (1%)	67 (1%)
**Complete cases**		
	550 (90%)	459 (87%)
**LVI positive/LVI negative**		
	90/523 (15%/85%)	92/433 (18%/82%)
