# Supplementary material for: Noninvasive Staging of Lymph Node Status in Breast Cancer Using Machine Learning: External Validation and Further Model Development
Source: JMIR Cancer. 2023 Nov 20;9:e46474. doi: 10.2196/46474 (PMC10696498; doi:10.2196/46474)
Supplement: Multimedia Appendix 6 [file cancer_v9i1e46474_app6.pdf]

Table S4. Patient and tumor characteristics in Cohort I.
The number of missing values is shown for non-complete case variables.

	All patients (n=761)	N0 (n=497)	N+ (n=264)
**Age** (years), median (range)			
	65 (24-92)	66 (33-91)	64 (24-92)
**Menopausal status**			
Premenopausal			
	128 (18%)	70 (15%)	58 (23%)
Postmenopausal			
	591 (82%)	397 (85%)	194 (77%)
Missing			
	42	30	12
**Mode of detection**			
Mammographic screening			
	447 (59%)	315 (63%)	132 (50%)
Symptomatic presentation			
	314 (41%)	182 (37%)	132 (50%)
**Tumor size** (mm), median (range)			
	15 (0.5-50)	13 (0.5-50)	18 (0.9-50)
Missing			
	1	0	1
**Multifocality**			
Absent			
	575 (76%)	402 (81%)	173 (66%)
Present			
	186 (24 %)	95 (19%)	91 (34%)

**Histological type**			
No special type (NST)			
	613 (81%)	397 (80%)	216 (82%)
Lobular			
	93 (12%)	57 (11%)	36 (14%)
Other invasive, including mixed types			
	55 (7%)	43 (9%)	12 (5%)
**NHG**			
I			
	186 (25%)	136 (29%)	50 (19%)
II			
	346 (47%)	224 (47%)	122 (47%)
III			
	221 (30%)	130 (27%)	91 (35%)
Missing			
	8	7	1
**ER status**			
Negative (< 1%)			
	67 (9%)	52 (11%)	15 (6%)
Positive (≥ 1%)			
	692 (91%)	443 (89%)	249 (94%)
Missing			
	2	2	0
**PR status**			
Negative (< 1%)			
	119 (16%)	87 (18%)	32 (12%)
Positive (≥ 1%)			
	640 (84%)	408 (82%)	232 (88%)
Missing			
	2	2	0

**HER2 status**			
Negative			
	618 (88%)	(89%)	209 (86%)
Positive			
	83 (12%)	(11%)	33 (14%)
Missing			
	60	38	22
**Ki67** (%), median (range)			
	15 (0-92)	14 (0-92)	17 (1-81)
Missing			
	42	28	14
**Lymphovascular invasion**			
Absent			
	523 (85%)	386 (93%)	137 (69%)
Present			
	90 (15%)	27 (7%)	63 (32%)
Missing			
	148	84	64
