# Supplementary material for: Noninvasive Staging of Lymph Node Status in Breast Cancer Using Machine Learning: External Validation and Further Model Development
Source: JMIR Cancer. 2023 Nov 20;9:e46474. doi: 10.2196/46474 (PMC10696498; doi:10.2196/46474)
Supplement: Multimedia Appendix 7 [file cancer_v9i1e46474_app7.pdf]

Table S5. Patient and tumor characteristics in Cohort III.
The number of missing values is shown for non-complete case variables.

	All patients (n=525)	N0 (n=401)	N+ (n=124)
**Age** (years), median (range)			
	66 (29-91)	66 (29-89)	65 (34-91)
**Menopausal status**			
Premenopausal			
	70 (14%)	51 (14%)	19 (17%)
Postmenopausal			
	415 (86%)	321 (86%)	94 (83%)
Missing			
	40	29	11
**Mode of detection**			
Mammographic screening			
	318 (61%)	252 (63%)	66 (53%)
Symptomatic presentation			
	207 (39%)	149 (37%)	58 (47%)
**Tumor size** (mm), median (range)			
	14 (1-50)	13 (4-50)	20 (1-50)
Missing (%)			
	9	8	1
**Multifocality**			
Absent			
	388 (75%)	310 (78%)	78 (63%)

Present			
	130 (25%)	85 (22%)	45 (37%)
Missing			
	7	6	1
**Histological type**			
No special type (NST)			
	389 (76%)	291 (74%)	98 (83%)
Lobular			
	91 (18%)	72 (18%)	19 (16%)
Other			
	41 (8%)	36 (9%)	5 (4%)
Missing			
	4	2	2
**NHG**			
I			
	133 (26%)	116 (29%)	17 (14%)
II			
	294 (57%)	216 (55%)	78 (64%)
III			
	95 (18%)	67 (17%)	28 (23%)
Missing			
	3	2	1
**ER status**			
Negative (< 1%)			
	34 (6%)	26 (6%)	8 (6%)
Positive (≥ 1%)			
	491 (94%)	375 (94%)	116 (94%)
**PR status**			
Negative (< 1%)			
	79 (15%)	63 (16%)	16 (13%)

Positive (≥ 1%)			
	444 (85%)	336 (84%)	108 (87%)
Missing			
	2	2	0
**HER2 status**			
Negative			
	496 (95%)	383 (96%)	113 (91%)
Positive			
	28 (5%)	17 (4%)	11 (9%)
Missing			
	1	1	0
**Ki67** (%), median (range)			
	21 (2-93)	20 (2-93)	29 (5-92)
Missing			
	1	0	1
**Lymphovascular invasion**			
Absent			
	433 (82%)	353 (88%)	80 (65%)
Present			
	92 (18%)	48 (12%)	44 (35%)
