# Supplementary material for: Noninvasive Staging of Lymph Node Status in Breast Cancer Using Machine Learning: External Validation and Further Model Development
Source: JMIR Cancer. 2023 Nov 20;9:e46474. doi: 10.2196/46474 (PMC10696498; doi:10.2196/46474)
Supplement: Multimedia Appendix 9 [file cancer_v9i1e46474_app9.pdf]

Table S6. Model architecture. All models comprised an input layer of various sizes, one hidden layer, and a one-node output layer. The models were trained during 400 epochs. The loss function was binary cross-entropy.

	Learning rate	Number of hidden nodes	L2 regularization	Dropout
**N-LVI_present[I]**				
	0.05	15	None	0.4
**N-LVI_imputed[I]**				
	0.01	6	L2(0.001)	0
**N-LVI_absent[I]**				
	0.04	10	None	0.4
**N-LVI_absent[II]**				
	0.009	8	None	0.5
**LVI model**				
	0.15	7	None	0.1

Abbreviations:

N, nodal

LVI, lymphovascular invasion
