# Supplementary material for: Noninvasive Staging of Lymph Node Status in Breast Cancer Using Machine Learning: External Validation and Further Model Development
Source: JMIR Cancer. 2023 Nov 20;9:e46474. doi: 10.2196/46474 (PMC10696498; doi:10.2196/46474)
Supplement: Multimedia Appendix 10 [file cancer_v9i1e46474_app10.pdf]

Table S7. Sensitivity, specificity, and false negative rate for the N models. The models N-LVI_present^{I}, N-LVI_imputed^{I}, and N-LVI_absent^{I} were evaluated in Cohort II (n=18 633), and the model N-LVI_absent^{II} was evaluated in the test cohort (n=3 727) of Cohort II.

	N-LVI_present^{I}	N-LVI_imputed^{I}	N-LVI_absent^{I}	N-LVI_absent^{I} (recalibrated)	N-LVI_absent^{II}
**Sensitivity**					
	91%	90%	90%	92%	91%
**Specificity**					
	25%	27%	24%	20%	30%
**FNR**					
	9%	9%	10%	8%	9%

Abbreviations:

N, nodal

LVI, lymphovascular invasion

FNR, false negative rate
