# Supplementary material for: Noninvasive Staging of Lymph Node Status in Breast Cancer Using Machine Learning: External Validation and Further Model Development
Source: JMIR Cancer. 2023 Nov 20;9:e46474. doi: 10.2196/46474 (PMC10696498; doi:10.2196/46474)
Supplement: Multimedia Appendix 12 [file cancer_v9i1e46474_app12.pdf]

Figure S5. Calibration of the N status model N-LVI_absent^{II} in the test cohort of Cohort II.

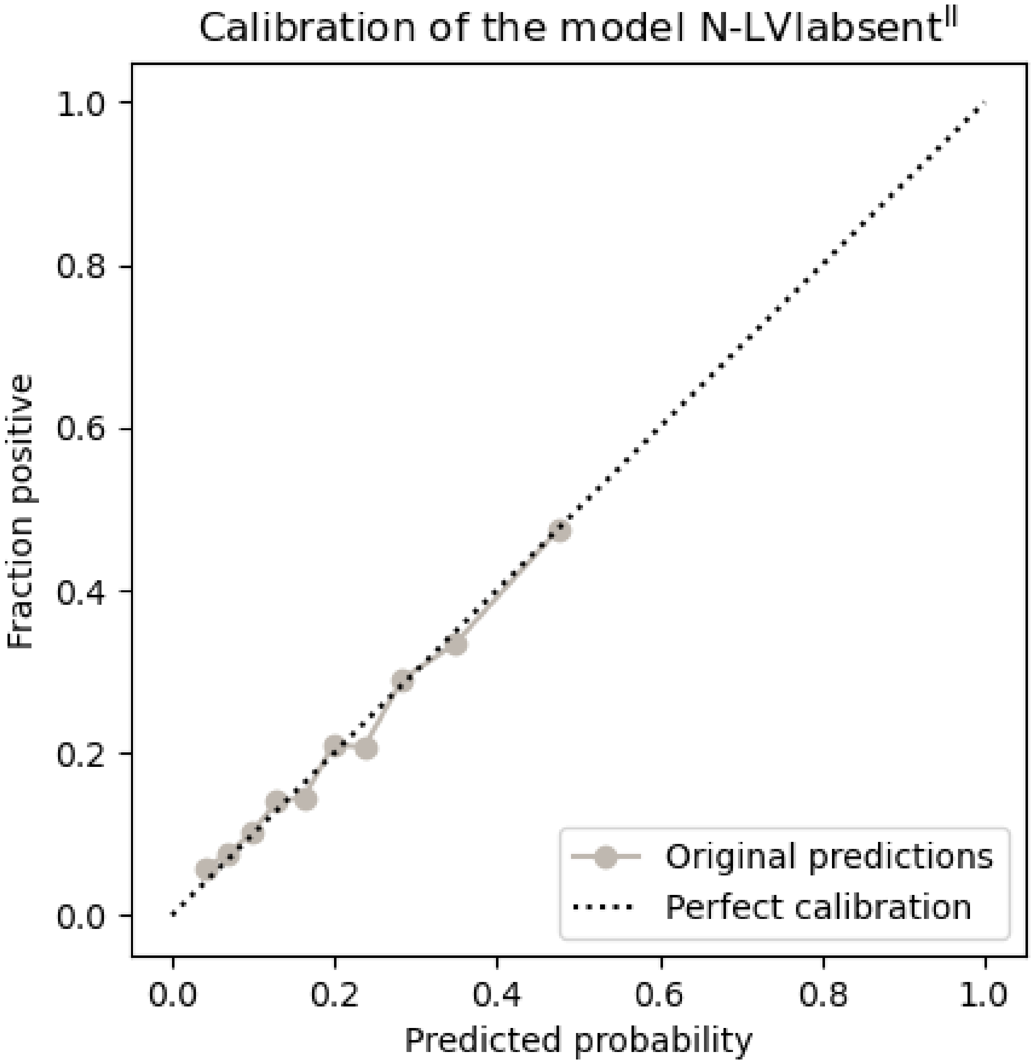

Abbreviations:

N, nodal

LVI, lymphovascular invasion
