# Supplementary material for: Noninvasive Staging of Lymph Node Status in Breast Cancer Using Machine Learning: External Validation and Further Model Development
Source: JMIR Cancer. 2023 Nov 20;9:e46474. doi: 10.2196/46474 (PMC10696498; doi:10.2196/46474)
Supplement: Multimedia Appendix 13 [file cancer_v9i1e46474_app13.pdf]

Figure S6. Standardized decision curve analysis for the original predictions of model N-LVI_absent[I]. The black horizontal line represents the scenario of all patients being diagnosed as node-negative; hence, no SLNB is performed. The colored function represents the diagnosis by the model. The golden, dashed, vertical line separating the lighter color from the darker shows the threshold for FNR < 10%. When all patients are considered node-positive and diagnosed through SLNB, the standardized net benefit is, by definition, zero. Note that the darker, colored area does not represent the patients spared from surgery. Rather, it displays the standardized net benefit of the model where FNR < 10%.

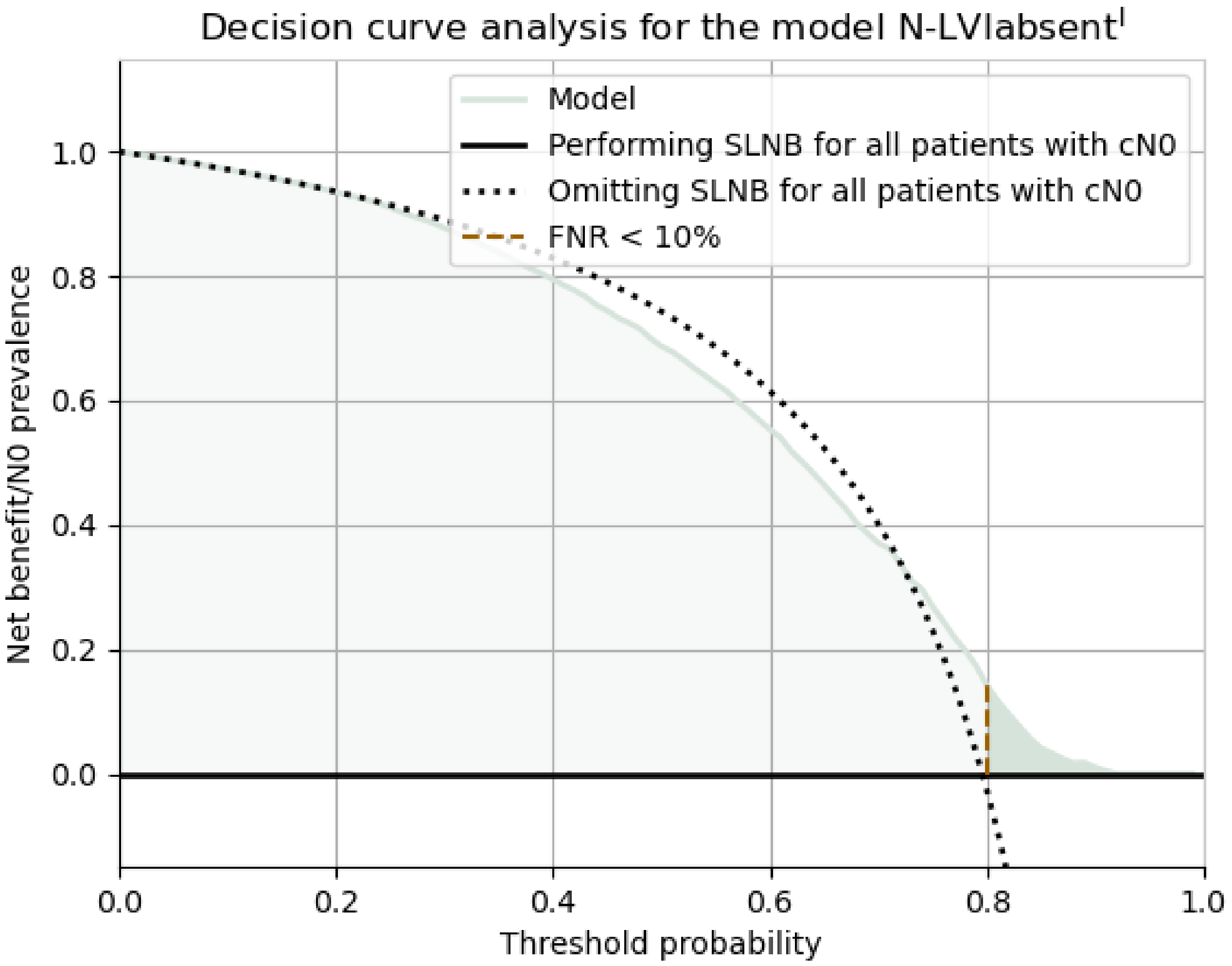

Abbreviations:

cN0, clinically node negative

FNR, false negative rate

SLNB, sentinel lymph node biopsy
